# Effects of a new nutraceutical combination on cognitive function in hypertensive patients

**DOI:** 10.1186/s12979-017-0113-4

**Published:** 2018-02-07

**Authors:** Giuseppe Giugliano, Alessia Salemme, Sara De Longis, Marialuisa Perrotta, Valentina D’Angelosante, Alessandro Landolfi, Raffaele Izzo, Valentina Trimarco

**Affiliations:** 1 Hypertension Research Center; Department of Advanced Biomedical Sciences, Federico II University, Naples, Italy; 2 Federico II University, Naples, Italy; 3 Hypertension Research Center; Department of Translational Medical Sciences, Federico II University, via Pansini 5, 80131 Naples, Italy; 4 Department of Angiocardioneurology and Translational Medicine, IRCCS Neuromed, Pozzilli, Isernia Italy; 5 Hypertension Research Center; Department of Neurosciences, Federico II University, Naples, Italy

**Keywords:** Nutraceutical, Neuropsychological evaluation, Arterial stiffness

## Abstract

**Background:**

Chronic increased arterial blood pressure has been associated with executive dysfunction, slowing of attention and mental processing speed, and later with memory deficits. Due to the absence of a concrete therapeutic approach to this pathophysiological process, in the last decades there has been an increasing interest in the use of nutraceuticals, especially those with antioxidant properties, which own strong neuroprotective potential, that may help to improve cognitive function and to delay the onset of dementia.

**Results:**

We evaluated the effects of the treatment with a new nutraceutical preparation containing different molecules with potent antioxidant properties (AkP05, IzzeK®) and placebo on a cohort of thirty-six hypertensive patients. At baseline, neuropsychological evaluation, arterial stiffness and biochemical parameters of the subjects were comparable. After 6 months of treatment, there was a significant reduction of the augmentation index in the AkP05-treated group. Moreover, the measurement of cognitive function, evaluated with MoCA test and Word Match Testing, showed a significant improvement in patients receiving the active treatment. In addition, the group treated with nutraceutical reached a better Stroop test score, while subjects that received placebo did not showed any improvement. Finally, a positive relationship between SBP variation and the psychometric assessment with the EQ-VAS scale was observed only in the active treatment group.

**Conclusions:**

In this study, we demonstrated that the therapy with a new nutraceutical preparation is able to significantly increase the scores of important neuropsychological tests in hypertensive patients already on satisfactory blood pressure control. Although future studies are needed to better characterize the molecular mechanisms involved, these results candidate the new nutraceutical combination as a possible therapeutic strategy to support the cerebrovascular functions and delay the onset of dementia in hypertensive patients.

## Background

One of the most common neurological disorders in the elderly is represented by a progressive and unremitting deterioration of cognitive functions, a condition globally referred to as dementia [1, 2]. In consideration of the lack of effective treatments and the demographic conditions shifting the population toward older adults, the incidence of dementia is predicted to exponentially increase in the future. Basically, for the vast majority of cases, cognitive impairment can be ascribed to two main causes: Alzheimer’s Disease (AD) and cerebrovascular diseases. Although it has been generally thought that AD represents those cases of dementia related to genetic alterations that leads to an increased production of the Aβ (amyloid β-peptide) and accumulation of plaques, we are now aware that a clear-cut distinction of AD and cerebrovascular diseases is not always found in the clinical practice, having patients a mixture of both pathologies [3]. On this notice, experimental models have shown that, even when no evident increase of Aβ production does exist, alterations of cerebrovascular homeostasis can determine a failure in the mechanisms responsible for the clearance of Aβ from the brain [4–6]. Thus, the definition of vascular cognitive impairment (VCI) predicts to recapitulate a wide range of cognitive deficits caused by vascular factors, and contributing to the development of later dementia, a condition where cognitive decline is irreversible and impairs even day-to-day functioning [7]. It is generally accepted that, whichever the primary cause, the outcome toward dementia and/or AD is a stage where no strategy is available to improve or at least counteracts the symptomatic manifestations of the disease.

Hypertension, a highly prevalent disease, representing nowadays one of the most impacting cause of disease burden and disability, is the major risk factor for cerebrovascular diseases [1]. Chronic increased arterial blood pressure has been associated with executive dysfunction, slowing of attention and mental processing speed, and later with memory deficits. Similarly, experimental models of hypertension allowed to unravel several pathophysiological mechanisms involved in this deleterious association [4, 8–10]. In addition, the intertwining of cerebrovascular diseases and AD can be also inferred from the neuropathological hallmarks of the two conditions, showing amyloid plaques and neurofibrillary tangles but also microvascular and ischemic lesions.

Taken together the neuropathological traits of brains affected by dementia have suggested over years the existence of an oxidative and inflammatory burden, deriving from the molecular lesions identified. Recent evidence suggests that immune and inflammatory processes significantly contribute to the progression of pathological hallmarks [11–15]. On a similar notice, inflammation and immune activation are clearly recognized as crucial aspects of hypertension and related target organ damage [16, 17].

The idea to counteract the inflammatory burden in early stages of cognitive deterioration and in those predisposing conditions, like hypertension and vascular risk factors, where pro-inflammatory and oxidative mechanisms have a considerable impact as well, raised an expanding area of research aimed at testing the potential beneficial effects of therapeutic strategies enhancing the global anti-oxidant and anti-inflammatory power. On this notice, several evidence in animal models support the use of combined nutraceutical supplementations in the prevention of cognitive decline [18–21]. At this regard different molecules with potent antioxidant properties, including Gingko biloba, extract of Bacopa monniera, Phosphatidylserine, a phospholipid component of the neuronal membrane, Green tea polyphenols and Epigallocathechin, although with mixed results, have shown to have positive effects on cognitive function also in humans [22–36].

According to these promising results, the present study was planned to asses in a double blind, parallel group versus placebo, the effects of a chronic treatment with a novel nutraceutical combination containing dry extract of Gingko biloba, Bacopa monniera, Green tea, phosphatidylserine and catechins on blood pressure, metabolic profile, arterial stiffness and cognitive function in hypertensive patients with a satisfactory blood pressure control obtained by an adequate antihypertensive therapy.

## Methods

### Study design

The study was conducted in accordance with the guidelines of the declaration of Helsinki and was approved by the Ethic Committee of the IRCCS Neuromed, Pozzilli (Isernia), Italy, which designed and registered the study protocol (ClinicalTrials.gov ID: NCT02572219). Written informed consent was obtained from each subject.

This was a randomized, double blind, placebo-controlled study with a 6-months follow-up, during which patients were randomized to receive placebo or active treatment of a nutraceutical preparation (AkP05, IzzeK®) containing: dry plant extract of *Bacopa monnieri* 300 mg + dry extract of *Ginkgo biloba* leaves 50 mg + Phosphatidylserine 25 mg + dry extract of green tea leaves 40 mg + Catechin 20 mg. Akademy Pharma produced the nutraceutical preparation. The company also produced the placebo, similar in appearance and organoleptic properties to the nutraceutical preparation.

Thirty-six patients were enrolled in the study between January 2017 and March 2017. Subjects of both sexes aged between 40 and 70 with diagnosis of essential hypertension were screened for assessment of the following exclusion criteria. Exclusion criteria were any of the following: previous cardiac or cerebrovascular event; heart failure; diabetes mellitus; history of atrial fibrillation or other severe arrhythmias; chronic kidney disease (defined as serum creatinine levels >1.4 mg/dL); pre-existing psychiatric disorders; neurodegenerative diseases such as multiple sclerosis, amyotrophic lateral sclerosis, Parkinson’s disease, early onset/genetic Alzheimer’s disease, neuromuscular pathologies, epilepsy; diagnosis of dementia. In addition, patients requiring any pharmacological treatment beyond antihypertensive drugs or with intolerance to nutraceutical compounds, pregnant women and women planning to conceive were also excluded from the study. Only hypertensive patients on pharmacological therapy and with a satisfactory and stable blood pressure control were enrolled, in order to rule out the possibility of an interference of potential additional antihypertensive drugs on cognitive function.

Eighteen were assigned to the nutraceutical treatment group and 18 to the placebo group, according to a computer based randomization double-blinded scheme.

### Procedures

Clinical history, risk factors and current pharmacological therapies were assessed at the baseline evaluation. Smokers included current and former smokers. Hypertension was diagnosed if systolic arterial pressure exceeded 140 mmHg and/or diastolic arterial pressure exceeded 90 mmHg, or if the patient was in antihypertensive drugs. Systolic and diastolic BP were measured by standard sphygmomanometer after 5 min in the supine position, according to the guidelines of the European Society of Hypertension/ European Society of Cardiology [37].

All patients suitable for enrollment underwent 2 pre-randomization visits: at baseline and after 2 weeks of run-in period in order to assess the stable and satisfactory blood pressure control. At the end of the run-in period, all randomized patients underwent the following evaluations:

- *Neuropsychological evaluation:* a battery of neuropsychological tests was administered to profile specific aspects of cognitive domains such as associative memory, visual-spatial memory, working memory, attentive skills, and reasoning skills. Cognitive assessment was administered by a well-trained psychologist (A.S.). The specific tests used were: the Montreal Cognitive Assessment (MoCA); Word pairing learning test; Stroop test; Visual analogue scale (EQ-VAS). The execution of all the neuropsychological tests was completed always in the same order.
- *Evaluation of arterial stiffness* was obtained by measuring the augmentation index (AI) with the SphygmoCor pulse wave analysis system (AtCor Medical Pty. Ltd., Sydney, Australia).
- *Peripheral blood sampling* for assessment of glucose and lipid profile.

### Neuropsychological tests

#### Montreal cognitive assessment (MoCA)

This test was designed as a tool for screening of mild cognitive deterioration and has been validated as a gold standard evaluation in vascular-related dementias [38]. The specific cognitive subdomains assessed are the following: attention, executive functions, memory, language, visuospatial abilities. The MoCA administration time is 10 min. The maximum possible score is 30 points; a score equal to or greater than 26 is considered normal.

#### Word match testing

The standard procedure requires reading a list of 10 pairs of words in the fixed order at the rate of a pair every 2 s. Next, in a different order from the first reading, the first member of each pair is read, and the subject is asked to recall the second. This procedure is repeated for 3 times. 5 pairs of words’ associations are usually easier (i.e. back and forth), while the other 5 are difficult (i.e. explosion-stamp). The test evaluates the associative memory in simple voluntary learning conditions.

#### Stroop test

This is a good example of the interference effect on highly automated tasks such as reading. The subject is asked to read the words in the first test, to name the colors in the second and third tests. It is necessary to mark both the mistakes made by the subject, but also the time spent in each trial. Results of the test are provided as two scores: 1) number of errors (Stroop E); and 2) number corrected for time and referred to as interference time (Stroop T).

#### Visual analogue scale (EQ-VAS)

Visual analogue scale is the second part of the EQ-5D test, in which the subject is asked to mark health status on the day of the interview on a 20 cm vertical scale with end points of 0 and 100. There are notes at the both ends of the scale that the bottom rate (0) corresponds to “the worst health you can imagine”, and the highest rate (100) corresponds to “the best health you can imagine”.

Participants were allowed to take a 5 min break if necessary to minimize tiredness and maintain motivation. The average duration of the whole neuropsychological evaluation was about of 30 min. For the first 3 tests, the scores obtained were corrected for age and level of education, following correction matrices developed according to validation rules for the Italian population [39]. Adjusted scores were then converted to a five-point interval scale, from 0 to 4 equivalent scores (ES) [39]. The five-point interval scale was divided as follows: 0 = below average scores; 1 = low average scores; 2 = average scores; 3 = high average scores; 4 = above average scores [39].

### Arterial stiffness

AI, a validated index of arterial stiffness, was measured by applanation tonometry. To record the central pressure waveform, the indirect method of arterial tonometry was used. Briefly, pressure waveform was recorded at the left radial artery and, using the generalized transfer function, it was converted into a calculated central pressure waveform [40]. All measurements were performed with the SphygmoCor device and designated software (AtCor Medical Pty. Ltd., Sydney, Australia). SphygmoCor uses a high fidelity Millar strain-gauge transducer (Millar Instruments, Houston, TX) allowing for measurement of the first systolic peak (P1), the second systolic peak (P2), and the central pulse pressure (PP) from the calculated aortic waveform. AIx was then calculated as: AI (%) = (P2-P1)PP*100. All patients were maintained without smoking, alcohol or caffeine, starting from the night before the evaluation. All measurements were performed with the patient in supine position in a quiet temperature-controlled laboratory (26 ± 1 °C).

### Endpoints

The primary endpoint was the effect of the two treatments (placebo vs active compound) on the score obtained at the Montreal Cognitive Assessment (MoCA) test. Secondary endpoints were the effects on other neuropsychological tests, arterial stiffness, as evaluated by SphygmoCor technology, and metabolic parameters.

### Statistical analysis

Based on preliminary data and previous studies assessing the impact of hypertension on cognitive functions, we hypothesized that 50% of patients treated with the nutraceutical preparation would experience an increase of at least one equivalent score (ES) of MoCA test (primary endpoint) with respect to 10% of subjects assuming placebo.

On this assumption, a sample size of 17 subjects in each group would have provided a power of 80% with a two-tailed α-error of 5%. In addition, expecting a drop-out rate of about 5%, due to the documented good tolerability of nutraceutical therapies, the total number of patients enrolled was of 36 (18 in each group). Data were analyzed using SPSS (version 22.0; SPSS, Chicago, Illinois, USA) and expressed as number and percentages or means ± SD. Differences between the two groups at baseline were evaluated by independent sample t-test or chi-square test, as appropriate. A general linear model (GLM) for repeated measures with correction for treatment was performed to evaluate the treatment effect in both groups. Pearson correlation coefficients were used to assess correlations between changes in clinical variables and changes in neuropsychological tests. For all tests, a *p* value <0.05 was considered statistically significant.

## Results

All the 36 patients enrolled completed the entire protocol of the study. Patients included in the study were predominantly males (75%) with a mean age of 58.1 ± 7.2 years, mean body mass index of 26.9 ± 3.0, mean SBP of 134.4 ± 15.1, and mean DBP of 85.0 ± 10.7 mmHg. At baseline, the patients of the two arms were comparable for all clinical and metabolic characteristics, arterial stiffness, and neuropsychological evaluation (Table 1). Also antihypertensive treatments were not different between the two study arms (Table 1).

**Table 1:** Baseline characteristics according to the treatment protocol group

	Placebo (*n* = 18)	Active treatment (*n* = 18)	*p*
Age (yrs)	57.9 ± 6.7	58.2 ± 8.0	0.910
Males	14 (77.8)	13 (72.2)	0.700
Height (cm)	170.0 ± 9.2	170.2 ± 8.6	0.955
Weight (kg)	75.9 ± 11.0	80.2 ± 15.7	0.351
Body Mass Index (kg/m^2^)	26.3 ± 3.1	27.7 ± 2.8	0.161
SBP (mmHg)	133.3 ± 16.9	135.6 ± 13.5	0.666
DBP (mmHg)	84.1 ± 12.3	85.8 ± 9.1	0.636
Heart rate (bpm)	68.8 ± 6.8	66.4 ± 7.9	0.350
Total cholesterol (mg/dl)	198.2 ± 23.7	201.9 ± 27.5	0.667
HDL cholesterol (mg/dl)	47.6 ± 7.9	51.3 ± 7.2	0.144
LDL cholesterol (mg/dl)	129.2 ± 26.1	126.7 ± 30.4	0.789
Triglycerides (mg/dl)	107.1 ± 34.8	119.5 ± 27.9	0.245
Uric acid (mg/dl)	5.8 ± 1.1	5.4 ± 0.8	0.175
Serum creatinine (mg/dl)	0.94 ± 0.14	0.99 ± 0.20	0.402
Glycaemia (mg/dl)	95.7 ± 11.2	93.1 ± 7.5	0.407
MoCA test ES	1.94 ± 0.94	1.89 ± 1.08	0.870
Word Match Testing ES	2.00 ± 0.97	2.50 ± 1.20	0.178
Stroop test E ES	2.22 ± 0.88	1.67 ± 1.65	0.215
Stroop test T ES	2.67 ± 0.91	2.28 ± 1.67	0.392
EQ-VAS	65.3 ± 12.8	68.1 ± 12.8	0.520
Augmentation Index (%)	14.19 ± 10.00	18.59 ± 18.21	0.400
Antihypertensive therapy			
ACE-inhibitors	2 (11.1)	2 (11.1)	1.000
ATII receptor blockers	13 (72.2)	14 (77.8)	0.700
Beta-blockers	4 (22.2)	5 (27.8)	0.700
Calcium channel antagonists	7 (38.9)	6 (33.6)	0.729
Diuretics	9 (50.0)	9 (50.0)	1.000

Values are numbers (percentage) or means ± standard deviation

SBP = systolic blood pressure; DBP = diastolic blood pressure; HDL = high-density lipoprotein; LDL = low-density lipoprotein; MoCa = Montreal Cognitive Assessment; ES = equivalent score; EQ-VAS = EQ-5D test visual analogue scale

**Fig. 1:**
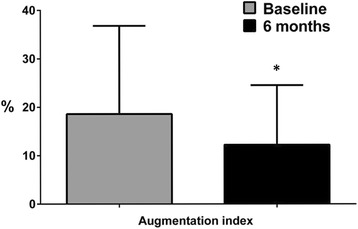
Augmentation index at baseline and after 6 months in the active treated group. * *p* = 0.028 for treatment effect (active treatment vs. placebo), GLM analysis

**Fig. 2:**
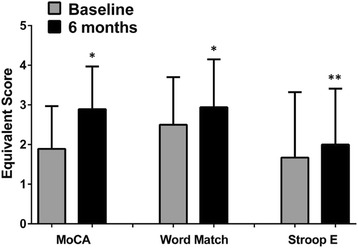
Scores of neuropsychological tests at baseline and after 6 months in the active treated group. * *p* < 0.050 for treatment effect (active treatment vs. placebo), GLM analysis. ** *p* = 0.050 for treatment effect (active treatment vs. placebo), GLM analysis

**Fig. 3:**
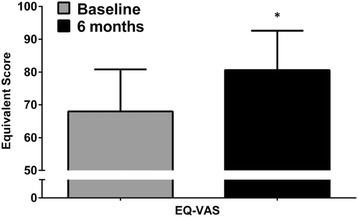
Visual analogue scale (EQ-VAS) score at baseline and after 6 months in the active treated group. * *p* < 0.001 for treatment effect (active treatment vs. placebo), GLM analysis

After 6 months, patients were subjected to the assessment of the same clinical and biochemical variables, as well as the cognitive profile. All the variables resulted unchanged in both placebo and active treatment groups, with respect to the baseline condition (Table 2), with the exception of AI which was significantly reduced in patients receiving the active nutraceutical compound (Table 2 and Fig. 1). When tested for the cognitive functions, the hypertensive patients in the active treatment group performed significantly better test than the placebo group, as compared to the baseline condition (Table 3 and Fig. 2). In particular, there was a significant improvement in the performance obtained at the MoCA test and Word Match Testing (Table 3 and Fig. 2) for patients receiving the nutraceutical active compound. With a trend toward significance, the same group of hypertensive patients executed the Stroop test with improved scores, whereas no variation was observed for patients receiving the placebo. The psychometric assessment with the EQ-VAS scale revealed a significant amelioration in the active treatment group (Table 3 and Fig. 3) but not in the control group receiving placebo (Table 3 and Fig. 3).

**Table 2:** Characteristics of patients at baseline and after 6 months of treatment

	Baseline	6-months	Treatment effect^a^
SBP (mmHg)			
Control group	133.3 ± 16.9	133.1 ± 15.7	0.132
Active treatment group	135.6 ± 13.5	130.6 ± 11.4	
DBP (mmHg)			
Control group	84.1 ± 12.3	83.1 ± 8.8	0.402
Active treatment group	85.8 ± 9.1	81.5 ± 8.5	
Heart rate (bpm)			
Control group	68.8 ± 6.8	68.2 ± 5.3	0.209
Active treatment group	66.4 ± 7.9	68.3 ± 8.3	
Total cholesterol (mg/dl)			
Control group	198.2 ± 23.7	210.8 ± 21.7	0.132
Active treatment group	201.9 ± 27.5	204.4 ± 23.6	
HDL cholesterol (mg/dl)			
Control group	47.6 ± 7.9	51.3 ± 11.9	0.740
Active treatment group	51.3 ± 7.2	54.3 ± 7.7	
LDL cholesterol (mg/dl)			
Control group	129.2 ± 26.1	138.9 ± 22.4	0.179
Active treatment group	126.7 ± 30.4	126.9 ± 25.0	
Triglycerides (mg/dl)			
Control group	107.1 ± 34.8	103.1 ± 35.0	0.946
Active treatment group	119.5 ± 27.9	115.9 ± 24.3	
Glycaemia (mg/dl)			
Control group	95.7 ± 11.2	96.6 ± 13.3	0.746
Active treatment group	93.1 ± 7.5	93.0 ± 8.0	
Augmentation Index (%)			
Control group	14.19 ± 10.00	14.69 ± 11.79	0.028
Active treatment group	18.59 ± 18.21	12.24 ± 12.30	

Values are means ± standard deviation

SBP = systolic blood pressure; DBP = diastolic blood pressure; HDL = high-density lipoprotein; LDL = low-density lipoprotein

^a^ = active treatment versus placebo, GLM analysis

**Table 3:** Characteristics of patients at baseline and after 6 months of treatment

	Baseline	6-months	Treatment effect^a^
MoCA test ES			
Control group	1.94 ± 0.94	1.83 ± 0.79	<0.001
Active treatment group	1.89 ± 1.08	2.89 ± 1.08	
Word Match Testing ES			
Control group	2.00 ± 0.97	1.72 ± 0.89	0.023
Active treatment group	2.50 ± 1.20	2.94 ± 1.21	
Stroop test E ES			
Control group	2.22 ± 0.88	1.94 ± 1.11	0.050
Active treatment group	1.67 ± 1.65	2.00 ± 1.41	
Stroop test T ES			
Control group	2.67 ± 0.91	2.22 ± 1.11	0.124
Active treatment group	2.28 ± 1.67	2.61 ± 1.42	
EQ-VAS			
Control group	65.3 ± 12.8	63.6 ± 12.3	<0.001
Active treatment group	68.1 ± 12.8	80.6 ± 12.0	

Values are means ± standard deviation

MoCa = Montreal Cognitive Assessment; ES = equivalent score; EQ-VAS = EQ-5D test visual analogue scale

^a^ = active treatment versus placebo, GLM analysis

**Fig. 4:**
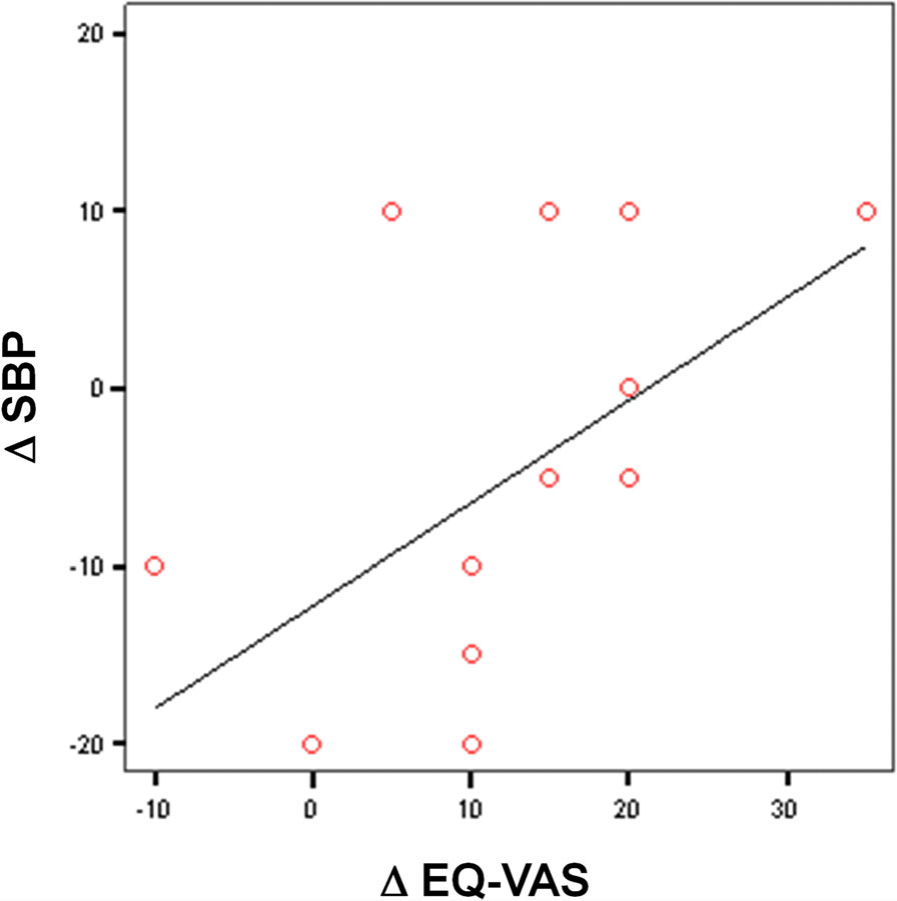
Correlation between change in systolic blood pressure and change in visual analogue scale (EQ-VAS) after 6 months of treatment in the active treated group

When we analyzed the potential correlations between changes of clinical and biochemical parameters with changes in neuropsychological scores obtained in the active treatment group, a positive relationship between SBP variation (from baseline to 6 months values) and EQ-VAS change was observed (*r* = 0.554, *p* = 0.017, Fig. 4).

## Discussion

The present randomized, double blind, placebo-controlled study demonstrated that a 6-month treatment with the novel nutraceutical preparation (AkP05, IzzeK®), containing dry plant extracts of *Bacopa monnieri*, *Ginkgo biloba* and Green tea, plus Phosphatidylserine and Catechins, significantly improved the cognitive performance at MoCA test, Word Match Testing, Stroop test, and EQ-VAS, in a population of hypertensive patients on satisfactory blood pressure control. In contrast, the hypertensive patients treated with the placebo composition, had no significant modification in the scores obtained at the various cognitive tests. Thus, the improvement observed in patients treated with the active nutraceutical preparation could be ascribed to positive effects on cognitive performance, ruling out the possibility of a placebo effect or potential habituation effects in performing the tests. In addition, hypertensive patients on active nutraceutical treatment displayed a significant improvement of arterial stiffness, as assessed by AI.

In this study, cognitive function was assessed accordingly to the most common measures used in general studies testing the impact of hypertension on cerebrovascular homeostasis. Basically, we tested how the nutraceutical compound under examination affected the most typically impaired cognitive domains of memory, executive function, and processing speed. The results obtained at the MoCA test after 6-months of treatment (active compound vs placebo), showed a significant improvement in the group of hypertensive patients receiving the nutraceutical preparation and no variation in the placebo group. Taking into consideration the fact that the battery of MoCA tests comprises a series of assessments capable to discriminate among different cognitive domains (memory and attention, visuospatial and executive functions, and language), we also evaluated more specific subdomains of cognition. Word-matching test highlighted a similar significant trend of improved learning abilities of the memory domain in patients receiving the active nutraceutical compound as compared to the placebo. The better performance in executive functions and processing speed was also confirmed when patients were subjected to the Stroop test, which showed enhanced functions in patients on active nutraceutical preparation. Lastly, the significantly increased score obtained in the VAS scale, indicative of patients’ awareness on their health status, was also suggestive that the benefit obtained with the 6-months treatment with the active nutraceutical compound was positively perceived by patients themselves.

Although several mechanisms may be involved in the improved overall cognitive function displayed by hypertensive patients treated with the nutraceutical compound, it could be envisaged that the concomitant reduction in arterial stiffness may be causally related. In fact, at the clinical level, indexes of aortic and large-artery stiffening are considered valuable predictors of cerebrovascular events and VCI [41, 42]. In this study, we observed that a 6-months treatment with the active nutraceutical compound induced a significant reduction in the AI, a parameter correlated with arterial stiffness. No significant variation of the AI was found in the group of patients receiving the placebo for 6 months. Although we failed to detect significant correlations between the changes in AI and any of the measured cognitive parameters, we could speculate that the improvement in arterial stiffness positively influenced cerebral circulation, eventually enhancing cognitive functions.

Chronic hypertension is also accompanied with hypertrophic remodeling of cerebral arteries and arterioles, a phenomenon well described in experimental models of hypertension and supported by evidence in patients. A critical aspect regarding cerebral circulation at the level of smaller arterioles consists in the disruption of the Blood-Brain Barrier (BBB), often leading to a potent inflammatory reaction, typically associated with the production of reactive oxygen species. A trait that can be commonly found in all the phenomena described above (hypertension, arterial stiffness, VCI and arterial stiffness) is the presence of a chronic low-grade inflammation, a process thought to contribute to pathogenesis of several diseases [14, 15]. Several experimental evidence suggests that the use of nutraceutical compounds can be effective in hampering cognitive decline in experimental models [18–21]. Thus, the possibility that hypertension could influence onset/progression of late-life dementia/sporadic AD by promoting neuroinflammatory processes through an increase in oxidative stress [43–46], accounts for the use of nutritional supplements with antioxidant action to counteract the development of cognitive decline in hypertensive patients. The nutraceutical preparation tested in the present study contains different molecules with potent antioxidant properties which, although with mixed results, have shown to have positive effects on cognitive function also in humans [22–32, 34], thus supporting the hypothesis that the improvement in cognitive function observed in our study is mediated by a reduction of the oxidative stress.

## Conclusions

The potential to counteract cognitive decline in hypertensive patients is of utmost importance. Although some studies [47–50] have shown little benefit from using antioxidant or anti-hypertensive drugs, they are often generally not well accepted as long-term preventive tools by the general population. By contrast, the use of dietary supplements and nutraceutical preparations is becoming more common and widespread, since they are often seen as a useful tool, associated with a healthy lifestyle, to deal with health problems that inevitably arise over time, in the presence of predisposing risk factors.

In this study, we demonstrated that the therapy with a novel nutraceutical preparation (AkP05, IzzeK®) containing *Bacopa monnieri*, *Ginkgo biloba*, Phosphatidylserine, Green tea and Catechins is able to significantly increase the scores of important neuropsychological tests such as the MoCA battery of tests, the Word Match Testing and Stroop test. The positive variation in the EQ-VAS was also suggestive that patients increased their perception of a better health status. The fact that an enhancement of performance in specific cognitive domain was obtained in patients already on satisfactory control of blood pressure levels, could reflect that the effect was exerted at the level of target organ damage, probably supporting cerebrovascular function by reducing oxidative stress at the level of brain microcirculation. Future pharmacological and pathophysiological studies are needed to better clarify the mechanisms underlying this effect.

## Abbreviations

AD: Alzheimer’s Disease
AI: augmentation index
Aβ: amyloid β-peptide
BBB: Blood-Brain Barrier
BP: blood pressure
DBP: diastolic blood pressure
ES: equivalent score
GLM: general linear model
MoCA: Montreal Cognitive Assessment
P1: first systolic peak
PP: pulse pressure
SBP: systolic blood pressure
VAS: Visual analogue scale
VCI: vascular cognitive impairment
